# Supplementary figures and images for: High-precision lighting for plants: monochromatic red laser diodes outperform LEDs in photosynthesis and plant growth
Source: Front Plant Sci. 2025 May 20;16:1589279. doi: 10.3389/fpls.2025.1589279 (PMC12129798; doi:10.3389/fpls.2025.1589279)

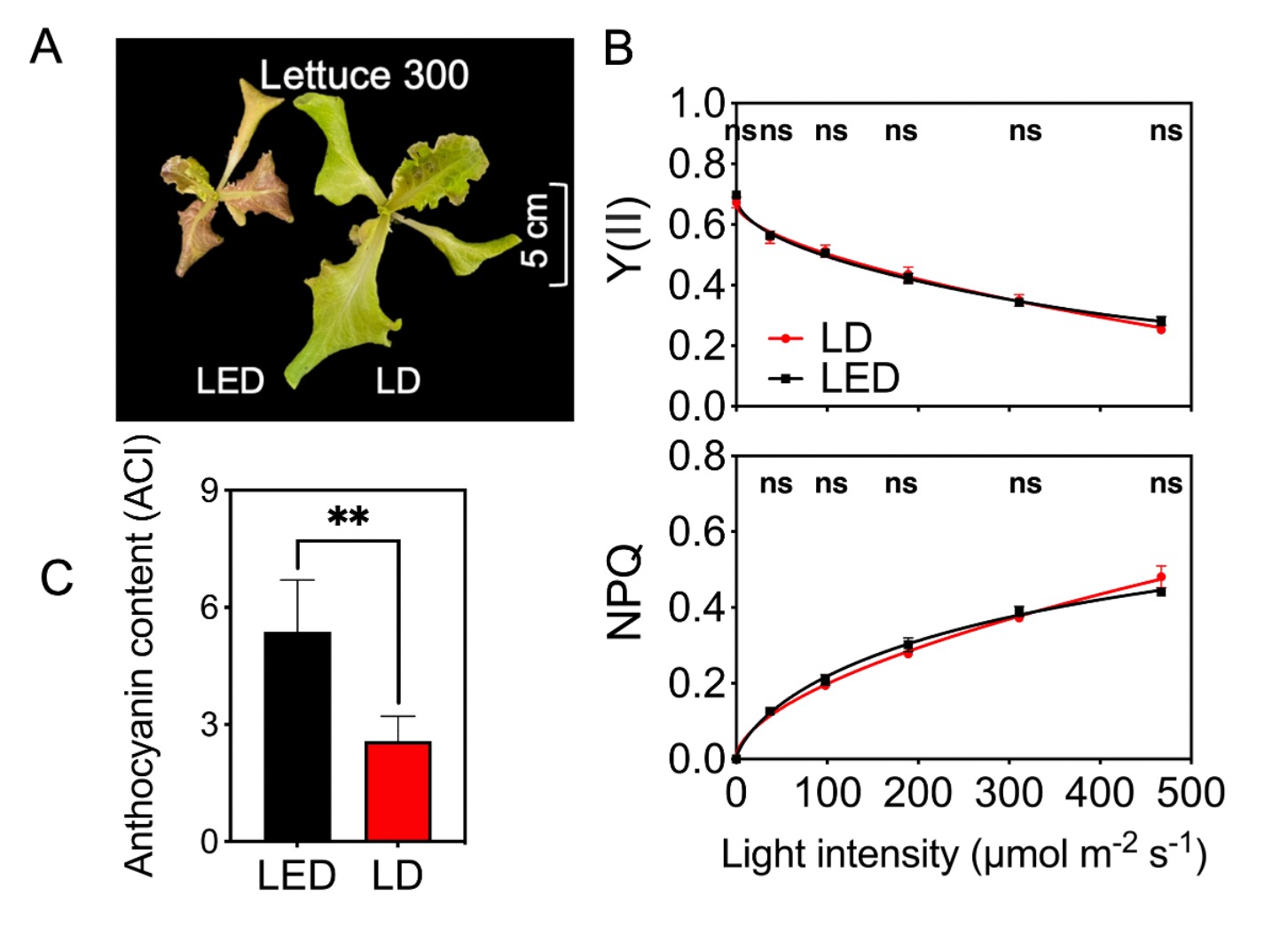

Supplement: Supplementary Figure 1 — Lettuce plants were grown under continuous LED 664 or LD 660 with a PPFD of 300 μmol m−2 s−1 for 12 d. (A) Representative image of a lettuce plant. (B) Response of the photosynthetic fluorescence parameters (Y(II) and NPQ) to different light intensities. (C) Accumulation of anthocyanin in the lettuce plants. ** indicates a significant difference at P < 0.01, ns indicates no significant difference according to the t test. The data are presented as the mean ± SE, n = 4. [file Image1.jpeg]
